# Architecture of CTPS filament networks revealed by cryo-electron tomography

**DOI:** 10.1016/j.yexcr.2024.114262

**Published:** 2024-09-19

**Authors:** You Fu, Chen-Jun Guo, Zhi-Jie Liu, Ji-Long Liu

**Affiliations:** a School of Life Science and Technology, ShanghaiTech University, Shanghai, 201210, China; b iHuman Institute, ShanghaiTech University, Shanghai, 201210, China; c Department of Physiology, Anatomy and Genetics, University of Oxford, Oxford, OX1 3PT, UK

**Keywords:** Cryo-electron microscopy, Cryo-electron tomography, CTPS, CTPS filament network, Cytoophidium, Metabolic filament

## Abstract

The cytoophidium is a novel type of membraneless organelle, first observed in the ovaries of *Drosophila* using fluorescence microscopy. *In vitro*, purified *Drosophila melanogaster* CTPS (dmCTPS) can form metabolic filaments under the presence of either substrates or products, and their structures that have been analyzed using cryo-electron microscopy (cryo-EM). These dmCTPS filaments are considered the fundamental units of cytoophidia. However, due to the resolution gap between light and electron microscopy, the precise assembly pattern of cytoophidia remains unclear. In this study, we find that dmCTPS filaments can spontaneously assemble *in vitro*, forming network structures that reach micron-scale dimensions. Using cryo-electron tomography (cryo-ET), we reconstruct the network structures formed by dmCTPS filaments under substrate or product binding conditions and elucidate their assembly process. The dmCTPS filaments initially form structural bundles, which then further assemble into larger networks. By identifying, tracking, and statistically analyzing the filaments, we observed distinct characteristics of the structural bundles formed under different conditions. This study provides the first systematic analysis of dmCTPS filament networks, offering new insights into the relationship between cytoophidia and metabolic filaments.

## 1 Introduction

In 2010, three independent laboratories discovered that CTP synthase (CTPS) could form large membraneless structures in *Drosophila* [1], bacteria [2], and budding yeast [3]. Due to their snake-like appearance, these structures were termed “cytoophidium” (plural, cytoophidia) [1]. Subsequently, CTPS cytoophidia have been observed in various species, including human [4,5], fission yeast [6–8], plants [9], zebrafish [10] and archaea [11], indicating that CTPS cytoophidia are evolutionarily conserved and could play significant roles in biological processes [12,13]. *Drosophila melanogaster* CTPS (dmCTPS) cytoophidia have been shown to extend the half-life of CTPS *in vivo* and are closely associated with the adipose body [14,15,16]. Additionally, CTPS cytoophidia are potentially involved in stress responses and cancer development [[17–23]].

CTPS catalyzes the final step in the de novo synthesis of cytidine-5′-triphosphate (CTP). It synthesizes CTP using UTP, ATP, and glutamine or ammonium as substrates under the regulation of GTP [24]. CTPS can act as 2′-deoxycytidine-5′-triphosphate (dCTP) synthase [25] and form filaments when bind to deoxyribonucleotides (dNTPs) [26]. Due to its importance in metabolic activities of life, CTPS is also considered a potential target for diseases such as cancer [27,28], tuberculosis [29–31], and autoimmune diseases [32,33].

In investigating the assembly mechanism and structure of dmCTPS cytoophidia, it was found that purified dmCTPS proteins can spontaneously assemble into filamentous structures *in vitro*, enhancing dmCTPS activity [34,35]. Human CTPS1 and CTPS2, as well as CTPS from yeast and bacteria, also form filaments that regulate their activity, suggesting evolutionary conservation of CTPS filamentation [36–39]. Many other metabolic enzymes have also been found to form filaments [40–48]. These filamentous structures, formed through the self-assembly of metabolic enzymes, are termed “metabolic filaments” [49,50]. This distinguishes them from other fibrous proteins, such as cytoskeletal proteins and amyloid proteins. With advancements in electron microscopy, the structures of an increasing number of metabolic filaments have been elucidated, revealing diverse regulatory roles and mechanisms. These discoveries have demonstrated that metabolic filaments add a new layer of regulation to metabolic enzymes.

dmCTPS filaments are considered the fundamental units of dmCTPS cytoophidia due to their filamentous structure. Although some studies have provided clues supporting this hypothesis, the precise assembly pattern and regulation of dmCTPS cytoophidia remain unclear [2,38]. dmCTPS cytoophidia are typically wider than 0.1 μm and can exceed 10 μm in length. Fluorescence microscopy, a common method for studying cytoophidia in situ, offers resolution up to several tens of nanometers. However, dmCTPS filaments are around 0.01 μm in width and up to 1 μm in length, making detailed analysis challenging with traditional fluorescence microscopy.

Electron microscopy based on single-particle analysis is commonly used to study CTPS filament structures, achieving subatomic resolution. However, this method is typically limited to sample smaller than 0.1 μm, making it unsuitable for direct analysis of cytoophidia. Cryo-electron tomography (cryo-ET) bridges the resolution gap between fluorescence microscopy and single-particle electron microscopy. It offers nanometer-level resolution and can image structures on a micron scale, enabling detailed analysis of cytoophidia.

In this study, we find that dmCTPS filaments spontaneously assemble into larger structural bundles under simulated substrate or product conditions *in vitro*, further forming extensive networks. After preparing cryo-samples, we use cryo-ET to resolve and reconstruct the network structure formed by dmCTPS filaments. By tracking, identifying, and statistically analyzing the filaments, we reveal structural characteristics and details of the network. We also identify similarities and differences between networks formed under two different conditions.

## 2 Results

### 2.1 Cryo-ET analysis of dmCTPS filament network

In our previous research, we discovered that the purified CTPS protein of *Drosophila* can spontaneously assemble into filament structures *in vitro* under the presence of substrates or products [34]. During this process, we incidentally observed that these filaments could assemble into larger complex structures.

Using negative-stain electron microscopy, we screened the sample conditions. In subsequent experiments, we identified using CTP to mimic the product condition of CTPS (referred as CTP-state) and UTP, ATP, the glutamine analog 6-diazo-5-oxo-norleucine, and the allosteric regulator GTP to simulate the substrate condition of CTPS (referred as DON-state). We prepared cryo-EM samples of the dmCTPS filament network in both CTP-state and DON-state: dmCTPS was first incubated with either substrates or products, then mixed with an equal volume of 10 nm colloidal gold particle solution, and finally rapidly frozen using a Vitrobot. The hole diameter of grid is 1.2 μm, and the center-to-center distance between holes is 1.3 μm. When we collect data, we select objects located within the ice layer of the hole, so the length and width of the study objects are usually below 1 μm. We collected cryo-ET data using a dose-symmetric data collection strategy. After 3D reconstruction and analysis of the data, we found that in both CTP-state and DON-state, dmCTPS filaments form structural bundles, which then further form networks.

**Fig. 1:**
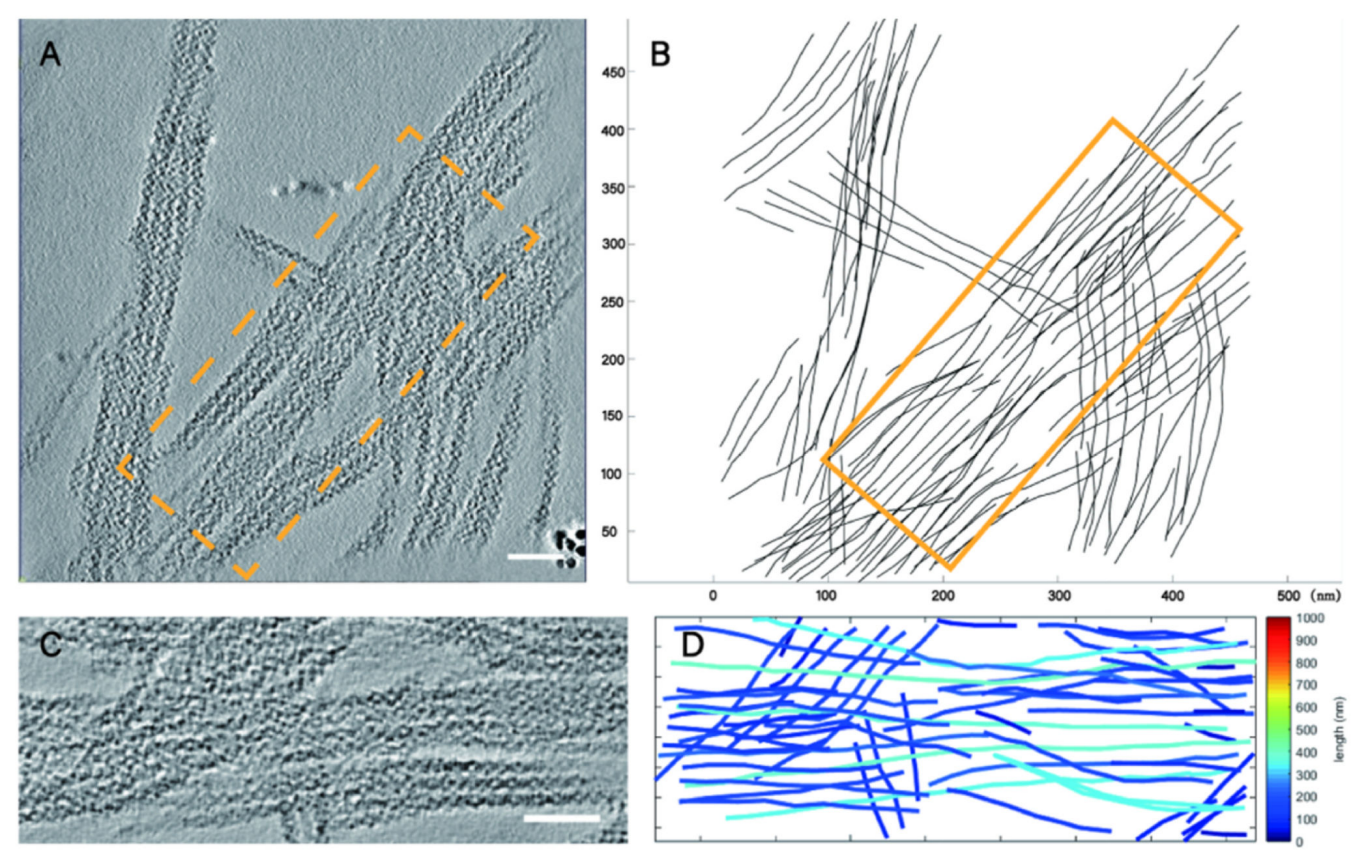
Selection and quantification of CTPS structural bundles. **A)** Representative three-dimensional reconstruction slice of the collected data. The yellow dashed box (150 nm wide, 400 nm long) indicates the selected region for structural bundle analysis. Scale bar: 50 nm. **B)** Schematic representation of the manually selected filament coordinates for A). The yellow solid box (150 nm wide, 400 nm long) within the figure shows the selected structural bundle used for subsequent quantitative analysis. **C)** Zoom-in view of the yellow box in A). Scale bar: 50 nm. **D)** Visualization for the filaments inside B). Each filament is colored by its lengths.

In the reconstructed 3D data (Fig. 1A), we manually selected and tracked filaments longer than 15 nm with continuous density, obtaining the spatial coordinates of the filaments (Fig. 1B). We focused our analysis on the bundles formed by these filaments.

For comparison, we processed the data within a defined spatial length and width, choosing the bundles with the highest number of filaments in the volume for analysis (Fig. 1C and D). Preliminary statistical analysis showed that a width of 150 nm and a length of 400 nm could maximally include structural bundles from different datasets, so we used these dimensions for statistical analysis of the structural bundle regions.

We randomly selected four datasets from both CTP-state and DON-state data (Fig. 1A and B) and compared and analyzed the spatial distribution of the filaments from multiple perspectives (Sup Fig. 1). The results showed that CTPS filaments primarily assembled in a parallel mode to form structural bundles, and these bundles would then form larger complexes in space, either in a roughly parallel or staggered manner.

### 2.2 dmCTPS filament network under CTP-state

**Fig. 2:**
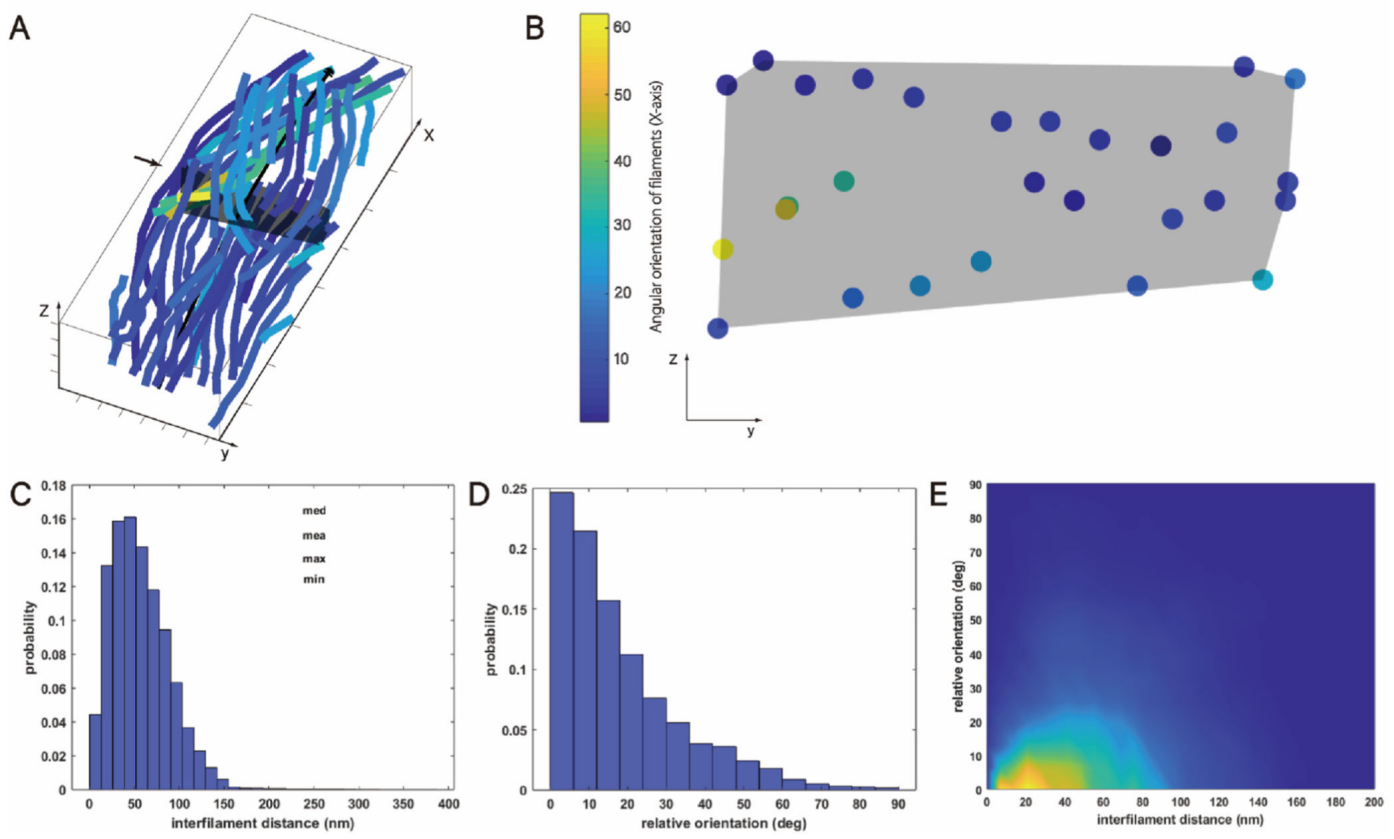
Quantitative analysis of CTP-state CTPS structural bundles. **A)** Three-dimensional visualization of a representative CTPS structural bundle in the CTP state. The black arrow parallel to the x-axis in the structural bundle marks the reference axis of the structural bundle. The black transparent polygon, indicated by the small black arrow perpendicular to the x-axis, indicates the cross-sectional position along this axis. The axes X, Y, and Z are also defined. **B)** Cross-sectional view of the CTPS structural bundle in the CTP state. This panel shows the distribution of individual filaments relative to the reference axis, corresponding to the position of the black transparent polygon in panel A. The color legend represents the local angular direction of the filaments relative to the reference axis. The Y and Z axes are shown. **C)** Statistical plot of distances (in nanometers) between adjacent filaments. This graph shows the distribution of distances between neighboring filaments. **D)** Statistical plot of relative local orientations (in degrees) between filaments. This graph illustrates the distribution of relative local orientations between filaments. **E)** Two-dimensional plot of filament-to-filament distances versus relative local orientations (in degrees).

The statistical analysis reveals that within the selected area (dimensions 150 nm × 400 nm), the average number of filaments per bundle in the CTP-state is 55 (Fig. 2A). CTPS filaments form bundles through mutual parallel and overlapping arrangements, with an average height of 46.75 nm. These bundles occupy an average cross-sectional area of 5413.5 nm^2^, with an average width of approximately 115 nm. On a representative cross-section of the sample (Fig. 2B), the filaments exhibit a characteristic pattern of wide lateral and thin vertical alignment, with a higher count of filaments in the lateral dimension (5-12) compared to the vertical dimension (1–4).

The minimum, maximum, median, and mean center-to-center distances between CTPS filaments were measured at 1.4 nm, 384.8 nm, 51.9 nm, and 56.8 nm respectively, indicating an overall approximately left-skewed normal distribution of filament spacing (Fig. 2C).

Analyzing the relative positional relationships of CTPS filaments in three-dimensional space reveals a median relative deviation direction of 13.4° and a mean of 18° between filaments (Fig. 2D). This suggests that within CTP-state bundles, despite some angular deviations between filaments, there is an overall tendency towards a consistent orientation.

Subsequently, we performed statistical analysis correlating filament distances (in nanometers) with their relative local orientations (in degrees) (Fig. 2E). The results indicate that under CTP-state, most filaments are arranged parallel with distances around 20 nm, with a smaller fraction arranged at approximately 40 nm intervals. Based on previous research, CTPS filaments are known to have a width of approximately 12 nm and a thickness of around 7 nm. Therefore, distances less than 10 nm suggest close proximity or direct contact between filaments. For example, in our sample, we observed instances where filaments appeared to branch, which might explain the minimal distance of 1.4 nm between them (Sup Fig. 2). This phenomenon could be due to estimation errors in identifying filament starting points during statistical analysis, causing one filament’s start point to be located within another filament. Alternatively, it might indicate the presence of two filaments sharing the same endpoint within the structural bundle. Additionally, the two-dimensional correlation plot reveals a minor presence of filament spacings at 60 nm and 80 nm. Filaments with angles less than 20° likely represent distant filaments within the same bundle, while those with angles greater than 20° may correspond to filaments from other bundles within the network.

### 2.3 dmCTPS filament network under DON-state

**Fig. 3:**
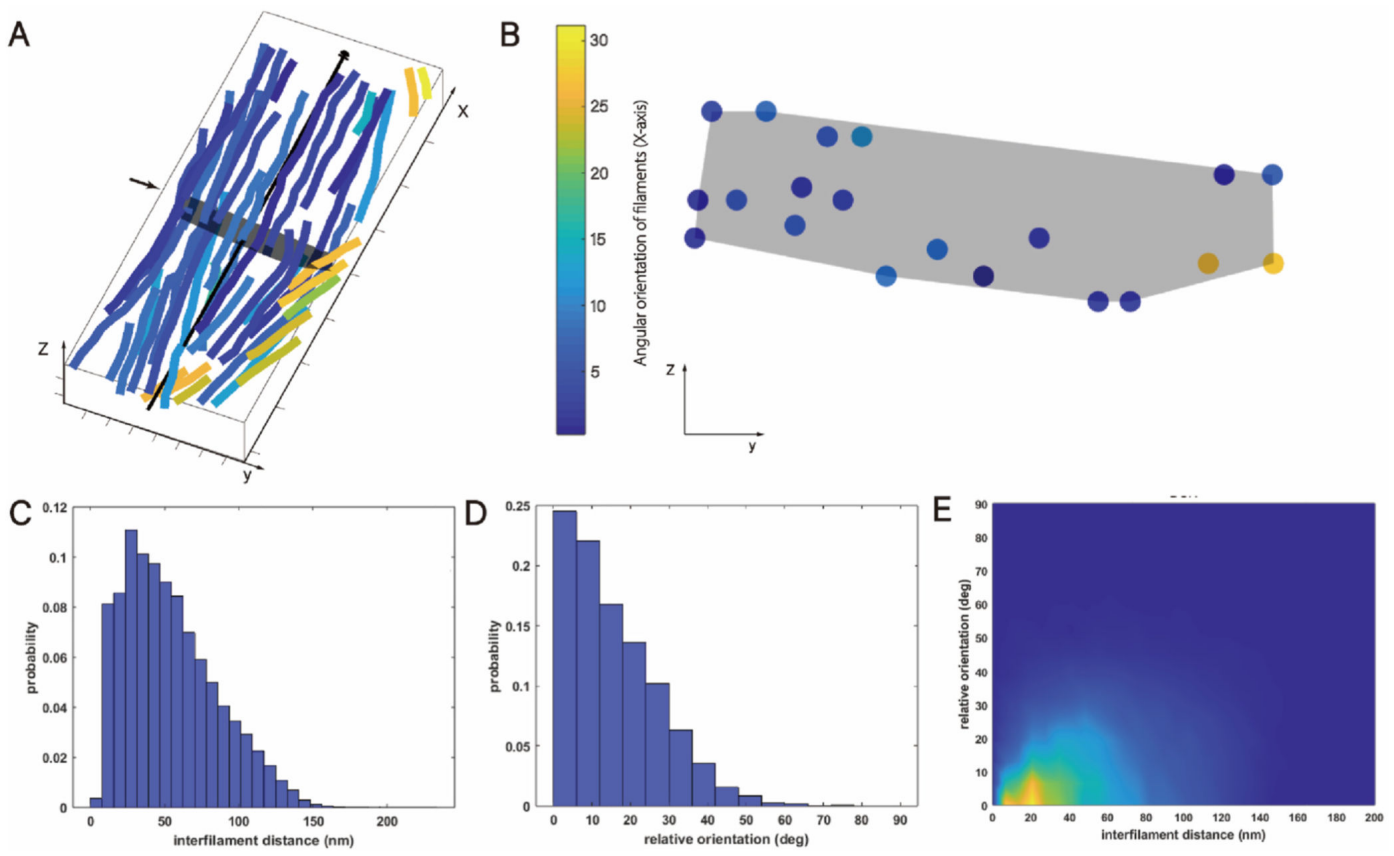
Quantitative analysis of DON-State CTPS structural bundles. **A)** Three-dimensional visualization of a representative CTPS structural bundle in the DON state. The black arrow parallel to the x-axis in the structural bundle marks the reference axis of the structural bundle. The black transparent polygon, indicated by the small black arrow perpendicular to the x-axis, indicates the cross-sectional position along this axis. The axes X, Y, and Z are also defined. **B)** Cross-sectional view of the CTPS structural bundle in the DON state. This panel shows the distribution of individual filaments relative to the reference axis, corresponding to the position of the black transparent polygon in panel A. The color legend represents the local angular direction of the filaments relative to the reference axis. The Y and Z axes are shown. **C)** Statistical plot of distances (in nanometers) between adjacent filaments. This graph shows the distribution of distances between neighboring filaments. **D)** Statistical plot of relative local orientations (in degrees) between filaments. This graph illustrates the distribution of relative local orientations between filaments. **E)** Two-dimensional plot of filament-to-filament distances versus relative local orientations (in degrees).

According to the results of statistical analysis, within the specified area (dimensions 150 nm × 400 nm), the average number of filaments per bundle in the DON state is 51, which is comparable to the CTP-state under same protein concentration. However, the structural bundles of DON-state exhibit an average height volume of 36.25 nm, occupying an average cross-sectional area of 3302.5 nm2, with an average width of approximately 91 nm. On representative cross-sections of the samples, filament distribution similarly displays characteristics of wide lateral and thin vertical alignment, with a higher number of filaments in the lateral dimension (approximately 6–7) compared to the vertical dimension (approximately 2–3) (Fig. 3A and B).

The minimum, maximum, median, and mean center-to-center distances between DON filaments and adjacent filaments are 1.1 nm, 231.7 nm, 48.3 nm, and 53.7 nm, respectively, also exhibiting an approximately left-skewed normal distribution, indicating a denser arrangement of filaments under DON conditions (Fig. 3C).

Analysis of the relative positional relationships between dmCTPS filaments in three-dimensional space shows a median relative direction (degrees) of 13° and a mean of 15.7°. This suggests that within the structural bundles in the statistical sample space, the DON-state structural bundles also tend to exhibit a consistent trend of filament aggregation, with smaller deviations in angles between filaments (Fig. 3D).

Correlating the distances between filaments (measured in nanometers) with their relative local directions (in degrees), it is observed that DON-state filaments predominantly arrange in parallel with distances around 20 nm (Fig. 3E). Compared to bundles in the CTP-state, filaments in the DON-state exhibit closer and more uniform spacing.

### 2.4 Comparison of two dmCTPS filament networks

To further contrast the spatial distribution of filaments within bundles under two different conditions, statistical analysis was conducted on relative filament angles, filament lengths, and filament curvatures. Initially, a second-order polynomial fitting was applied to coordinates of all filaments within the structure to establish a reference direction (axis) for the overall bundle. This fitting method helps capture the curvature and variations in filamentous structures, facilitating subsequent analysis and understanding of mechanical characteristics. Subsequently, by measuring and statistically analyzing the angle between each filament’s end-to-end vector and the reference direction, the distribution of relative filament axial angles can be obtained.

**Fig. 4:**
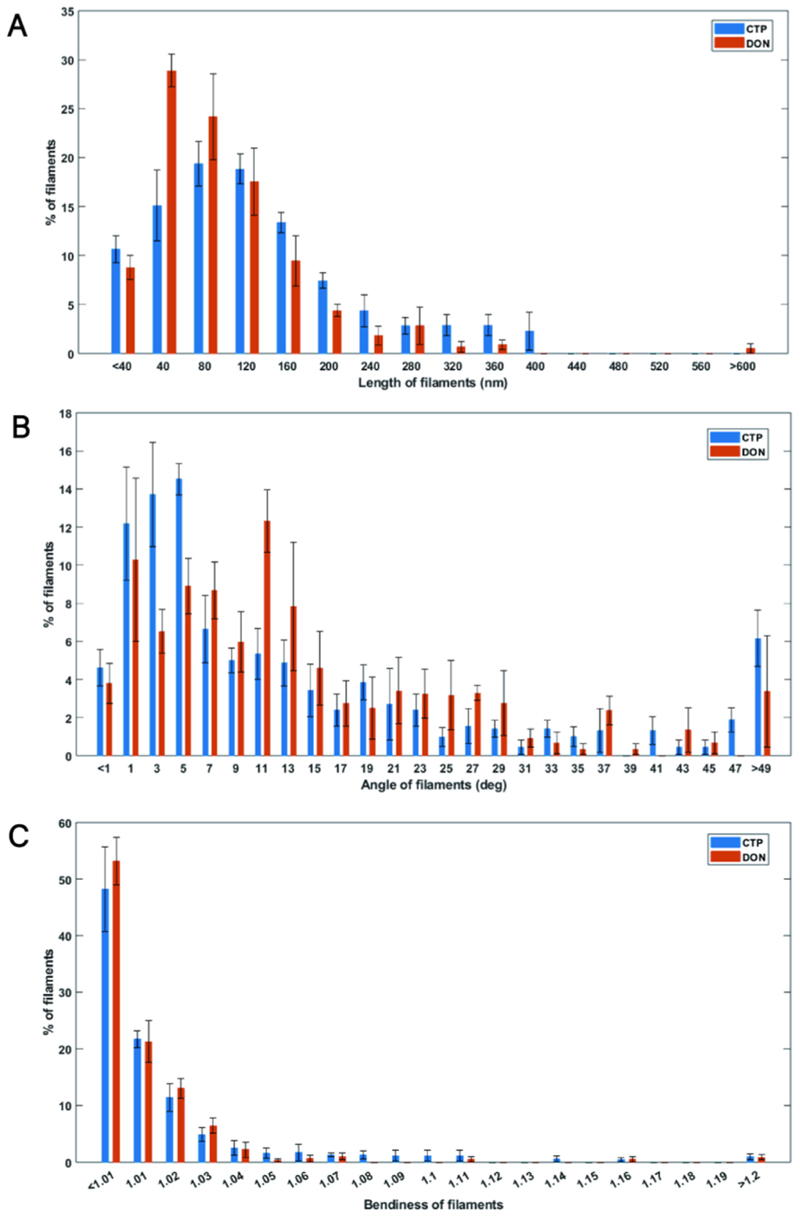
Comparison of filament characteristics in structural bundles between CTP and DON-States. **A)** Comparison of filament length distribution between CTP and DON states. This panel compares the distribution of filament lengths within the structural bundles in the CTP and DON states. **B)** Comparison of filament angle distribution relative to the reference axis between CTP and DON states. This panel contrasts the distribution of filament angles relative to the reference axis in the CTP and DON states. **C)** Comparison of filament curvature distribution between CTP and DON states. This panel examines the distribution of filament curvature in the structural bundles between the CTP and DON states.

In the statistical sample space, the average filament length under CTP-state was 149.3 nm, longer than the 119 nm observed under DON-state (Fig. 4A). Distribution analysis of individual filament lengths shows that both CTP and DON filament lengths are predominantly concentrated within 200 nm, accounting for 77.31 % and 88.82 % of the total, respectively, with a minority extending between 200 nm and 400 nm, accounting for 10.42 % and 10.66 %, respectively.

Comparison statistics of relative filament angles reveal that under both CTP and DON conditions, filaments within the same structural bundle exhibit similar orientations (Fig. 4B). Approximately half of the filaments deviate from the reference direction by less than 11°. Specifically, 56.6 % of CTP-state filaments are concentrated within 10°, and 85.46 % within 30°, while 50.79 % of DON-state filaments are within 11°, and 90 % within 30°. Although there are differences in distribution between CTP and DON conditions, with 39.69 % of CTP-state filaments and 25.46 % of DON-state filaments having deviations ≤ 5°, and 13.31 % for CTP-state and 21.82 % for DON-state between 9 and 13°, this suggests varying modes of filament binding or interaction within structural bundles under CTP and DON conditions. Specifically, filaments in CTP-state tend to align more axially, while filaments in DON-state exhibit greater relative axial angles.

Filament curvature was defined as the ratio of filament length to the distance between its two ends, where a curvature of 1 indicates a straight line, and values greater than 1 indicate curvature, with higher values indicating greater structural curvature. Approximately half of the filaments under CTP and DON conditions approximate straight lines, comprising 48 % for CTP-state and 53 % for DON-state (Fig. 4C). Curvature exhibited a negative correlation with relative abundance; higher curvature corresponded to fewer filaments. A minority of filaments exhibited significant curvature, with approximately 10 % of CTP filaments having curvatures above 1.05, and approximately 4 % of DON filaments having curvatures above 1.05.

In previous *in vitro* studies, the CTPS filaments resolved in the DON and CTP states exhibited different structures. Under the DON-state, the tetramer is in a close conformation with more tightly packed stacking, theoretically making DON-state filament less prone to bending [35]. On the other side, *in vivo* experiments have shown that the addition of DON not only promotes the formation of cytoophidia but also increases the proportion of highly curved, ring-shaped cytoophidia among all cytoophidia [4,51].

This is consistent with the results of our analysis, where single filaments in the DON-state indeed have less curvature. Additionally, larger relative axial angles may provide a possible explanation for the observed increase in the proportion of ring-shaped cytoophidia/all cytoophidia upon the addition of DON *in vivo*.

### 2.5 Filament distribution within the structural bundles

**Fig. 5:**
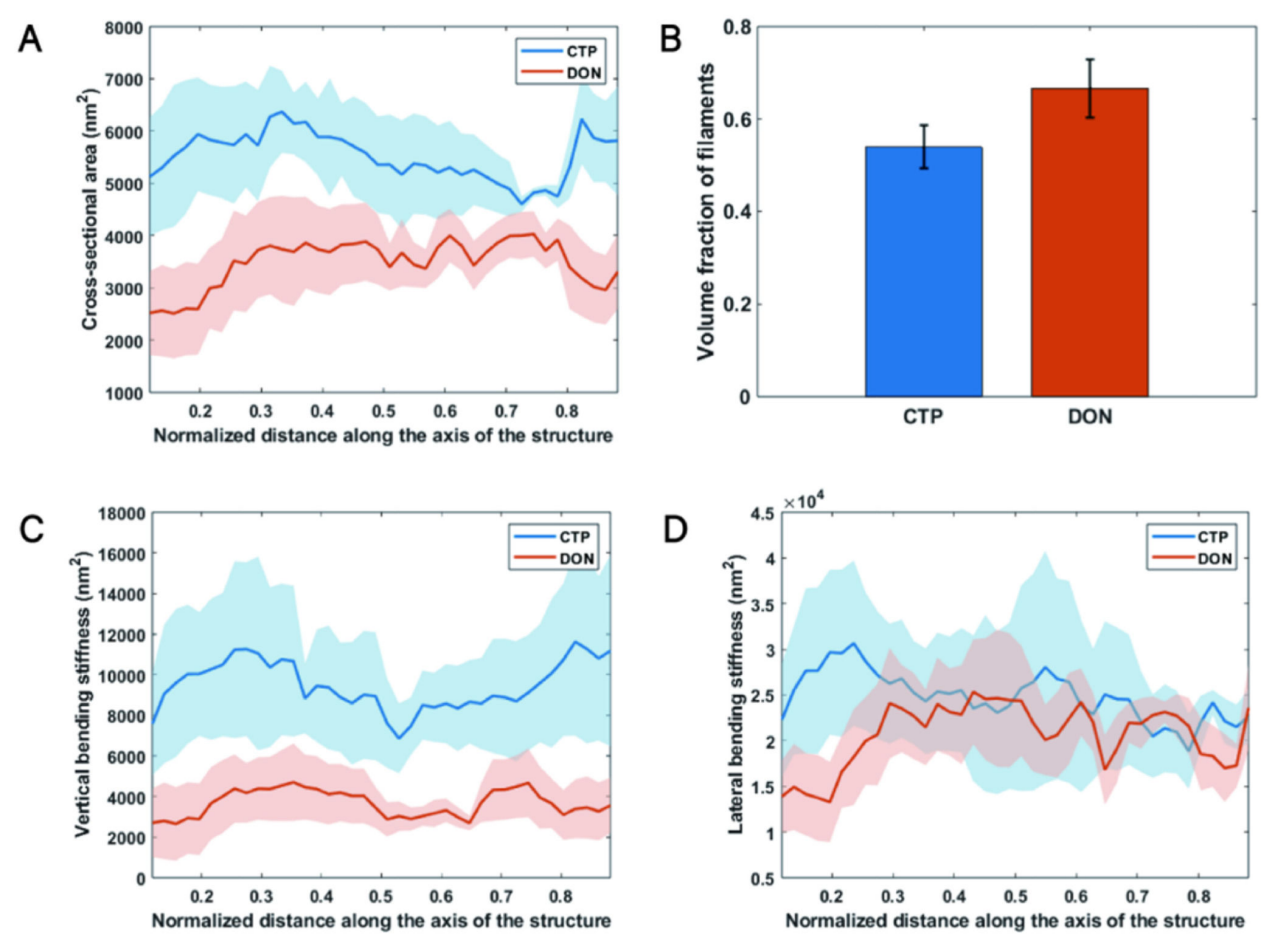
Comparison of structural bundles characteristics between CTP and DON states. **A)** Comparison of average cross-sectional area along the axis of the structural bundles. This panel compares the average cross-sectional area along the axis of the structural bundles between the CTP and DON states. Transparent outlines represent standard deviations. **B)** Histogram of volume ratios. This histogram illustrates the volume ratio between the total volume of filaments and the total volume of the structure, comparing the CTP and DON states. **C)** Comparison of average vertical stiffness along the axis of the structural bundles. This panel contrasts the average vertical stiffness along the axis of the structural bundles between the CTP and DON states. **D)** Comparison of average lateral stiffness along the axis of the structural bundles. This panel compares the average lateral stiffness along the axis of the structural bundles between the CTP and DON states. Transparent outlines represent standard deviations.

In addition, we calculated the distribution of filaments along the axis of the entire structure. Averages and tracking were performed along the axis of the entire bundle. By calculating the cross-sectional areas of structures under CTP and DON conditions, it was observed that the cross-sectional area under CTP conditions (ranging from 3986.57 to 7246.64 nm^2^) was greater than under DON conditions (ranging from 1645.23 to 4757.36 nm^2^) (Fig. 5A).

To measure structural density, we defined the volume ratio as the ratio of the total volume of all filaments to the total volume of the bundle. The CTP-state ratio was 0.54, while the DON-state ratio was 0.67, indicating that the structural density of bundles in DON-state is higher than in CTP-state (Fig. 5B). Specifically, under equal statistical spatial volumes, the number of filaments in DON conditions exceeds that in CTP conditions by 20 %.

Additionally, given that the constituents of the bundles are identical, we utilize the moment of inertia of the filaments with respect to the y-axis in the cross-section to describe the structure’s resistance to bending in the z-direction, which we refer to as the vertical bending stiffness. Stiffness of bundles under CTP and DON conditions was computed (Fig. 5C and D). It was found that the vertical bending stiffness of the structure under CTP conditions was 9138.44 nm^2^, compared to 3526.72 nm^2^ under DON conditions. Similarly, the transverse bending stiffness under CTP conditions was 23876.5 nm^2^, whereas under DON conditions it was 19361 nm^2^. This indicates that bundles in the CTP-state exhibit greater resistance to bending in both vertical and transverse directions compared to those in the DON-state. Specifically, in the vertical direction, the CTP-state demonstrates more than double the bending resistance of the DON-state, assuming equal filament strength.

Overall, these findings indicate that CTP filaments form a loosely packed parallel bundle structure with greater filament spacing and relative angles, yet exhibit strong overall resistance to external bending, particularly in the vertical direction. In contrast, DON structural bundles consist of filaments tightly packed in parallel, with smaller filament spacing and relative angles, but weaker overall resistance to external bending, especially in the vertical direction.

## 3 Discussion

Since the discovery of the cytoophidia in 2010, the lack of detailed structural knowledge has constrained investigations into its functionalities. CTPS metabolic filaments are considered fundamental building blocks of the CTPS cytoophidia, yet the mechanisms governing their further assembly remain unclear. In this study, we observed further assembly of dmCTPS metabolic filaments *in vitro* and utilized cryo-ET to analyze the complex structures formed under two different states.

**Fig. 6:**
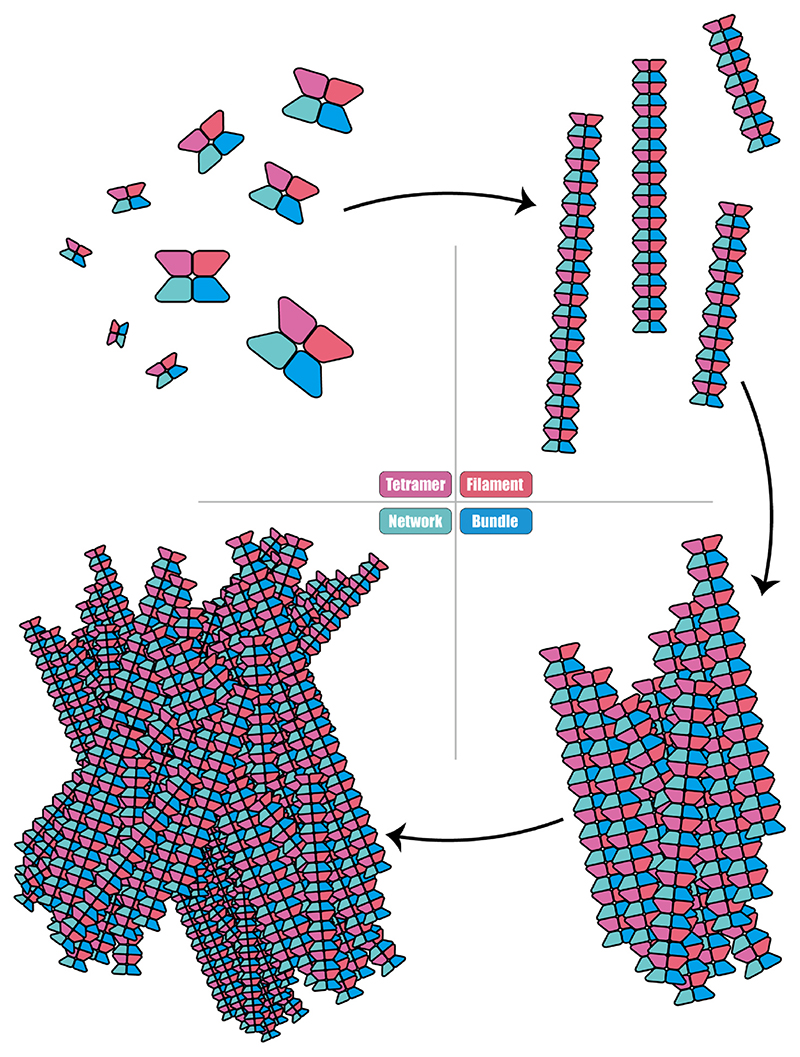
Model of CTPS filament networks assembly. With substrates ATP and UTP or products CTP, CTPS first forms tetramers, which then assemble into filaments. Multiple filaments are further assembled in an approximately parallel manner into a structural bundle. The structural bundles are then further assembled into larger network structure.

Our findings indicate that CTPS filaments predominantly assemble into structural bundles in a parallel configuration, further aggregating to form larger networked complexes (Fig. 6). The volumetric occupancy of CTPS filaments within these structural bundles, under both CTP-state and DON-state conditions, averages less than 70 %. This suggests that CTPS structural bundles are not densely packed but exhibit a degree of porosity, potentially facilitating ligand exchange during CTPS metabolic processes.

Further statistical analyses reveal distinct features of CTPS filament complexes under CTP-state and DON-state conditions (Sup Fig. 3). For instance, analyses of structural bundle stiffness indicate that structural bundles in the CTP-state exhibit greater resistance to bending. It has been observed that the arrangement of actin exhibits different patterns both *in vivo* and *in vitro*, corresponding to different types of functions, such as: lamellipodium (branched and crosslinked filament) and filopodium (bundle of parallel filaments) [52]. In our study, CTPS filaments show differentiated structural characteristics under different conditions *in vitro*. This suggests that CTPS filaments respond to environmental changes and have different types of functions *in vivo*.

Utilizing cryo-ET, this study provides new insights into the assembly of CTPS filament complexes. However, limitations include the sample preparation and manual selection of filaments, potentially introducing errors. During the preparation of cryo-electron microscopy samples, the specimen typically needs to form a thin liquid film on the grid, thereby controlling the sample thickness to below 200 nm. In this process, surface tension determines the spreading behavior of the liquid on the grid, affecting the uniformity and thickness of the ice, which may also impact the arrangement of CTPS filaments. Furthermore, to enhance the adhesion of the sample to the grid, plasma treatment and other methods are commonly used to hydrophilize the grid during the sample preparation process, which may also affect the arrangement of CTPS filaments. Thus, the assembly of dmCTPS complexes *in vitro* may differ from the physiological conditions *in vivo*, necessitating further research to validate these findings in cellular environments.

## 4 Methods

### 4.1 Protein purification and sample preparation

The protein was purified using the same method as before. A concentration of 50 μM CTPS was used to prepare the sample solution. For the DON-state and CTP-state samples, we added 60 μM DON, 2 mM ATP, 2 mM UTP, 2 mM GTP, and 10 mM magnesium ions or 2 mM CTP and 10 mM magnesium ions to the system, respectively. Ligands and protein are incubated at room temperature for 30 min. Subsequently, an equal volume of gold nanoparticles (Aurion Gold Nanoparticles – Carboxyl Functionalized-10nm) was added to the system. After thorough mixing, 3 μL of the mixture was applied onto a hydrophilized grid(No. M024-Au300-R12/13), blotted using a Vitrobot, and rapidly plunged into liquid ethane to obtain the cryo samples.

### 4.2 Cryo-ET data collection

Cryo-ET data was collected using a Titan Krios G4 300 kV electron microscope equipped with an energy filter and a Falcon4 detector (Thermo Fisher Scientific). The Tomography software facilitated the acquisition of tilt series. To achieve high-resolution reconstruction, a dose-symmetric tilt scheme [53] was used during data collection with a magnification of 85,000x, a pixel size of 1.22 Å, and a defocus of 2 μm. A tilt series spanning ±30° in 2° increments was collected, comprising 31 angles, with an electron dose of 4.5 e/Å^2^ per angle and a cumulative dose of 140 e/Å^2^ per tilt series.

### 4.3 Image preprocessing

Motion correction and Contrast Transfer Function (CTF) estimation were conducted on each image using Relion4.0 [54] to compensate for sample movement and focusing errors. The processed single images were then reassembled into new tilt series based on their respective tilt angle information.

### 4.4 Tilt-series alignment

Tilt series alignment was performed using IMOD [55] to correct for sample movement during tilting. A fourfold binned three-dimensional reconstruction was carried out using Weighted Back Projection (WBP). Three-dimensional data were processed with a twelvefold binning using IMOD, and image signal enhancement was performed using the ISONET [56] software to achieve better image contrast.

### 4.5 Filament identification and statistical analysis

Four distinct datasets for each of CTP and DON were randomly chosen for comprehensive analysis. Utilizing the Dynamo [57] software, filaments were meticulously identified through manual selection with the filament along axis module. Filaments exceeding 30 nm in length and exhibiting discernible continuous density were categorized as individual entities.

To facilitate further analysis, custom scripts were developed to convert the resulting coordinate files from.mat format to.txt, enabling seamless integration with the MATLAB-based Computational Toolbox [58]. This conversion allowed for a comprehensive quantitative analysis of the data.

Employing the cropping.m script, we extracted individual structural bundles, each measuring 150 nm by 400 nm. The Analysis_Filopodia.m script was then executed to precisely delineate the X-axis orientation of these bundles.

Visualization of the three-dimensional structure and cross-sectional profiles of the filaments was achieved using the Plots_Single_Cell_Filopodia.m script. This enabled us to calculate key statistical metrics, including the total filament count, average cross-sectional area, and cumulative volume for each structural bundle.

Finally, the Plots_Group_Cell_Filopodia.m script was engaged to scrutinize the interactions between CTP and DON. This analysis encompassed a range of parameters such as filament angles, lengths, and cross-sectional dimensions, providing a nuanced understanding of the interplay between these two components.

## Supplementary Material

Supplementary data to this article can be found online at https://doi.org/10.1016/j.yexcr.2024.114262.

Supplementary file

## Data Availability

Data will be made available on request.
