# Supplementary material for: Architecture of CTPS filament networks revealed by cryo-electron tomography
Source: Exp Cell Res. Author manuscript; Available in PMC 2026 Apr 10. (PMC7618999; doi:10.1016/j.yexcr.2024.114262)
Supplement: Supplementary file [file EMS213047-supplement-Supplementary_file.docx]

**Supplementary figure S1**


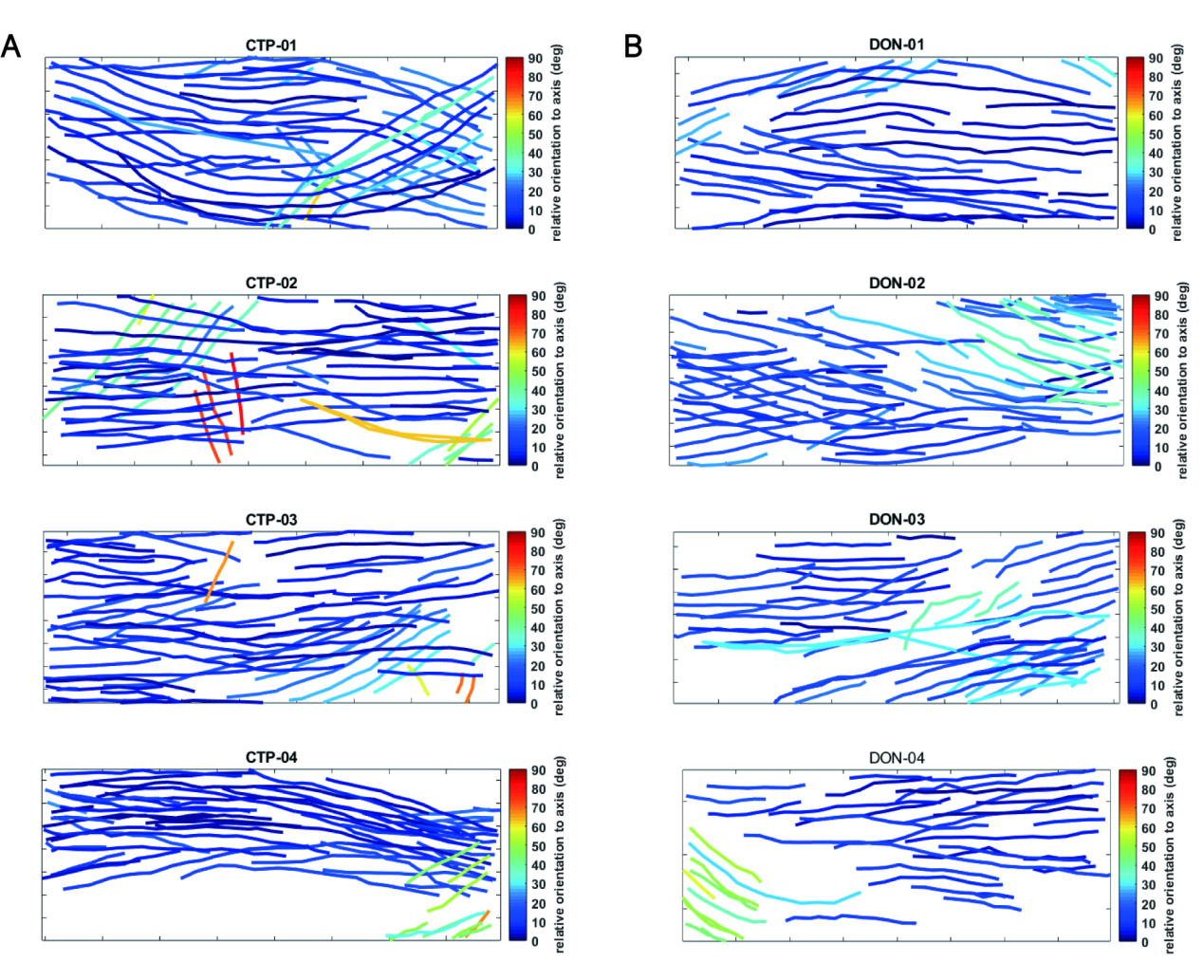


**Angular relationship between analysis data and direction axis**

**A.** Visualization of the analyzed data from CTP-state. **B.** Visualization of the analyzed data from DON-state. The color legend represents the local angular direction of the filaments relative to the axis.

**Supplementary figure S2**


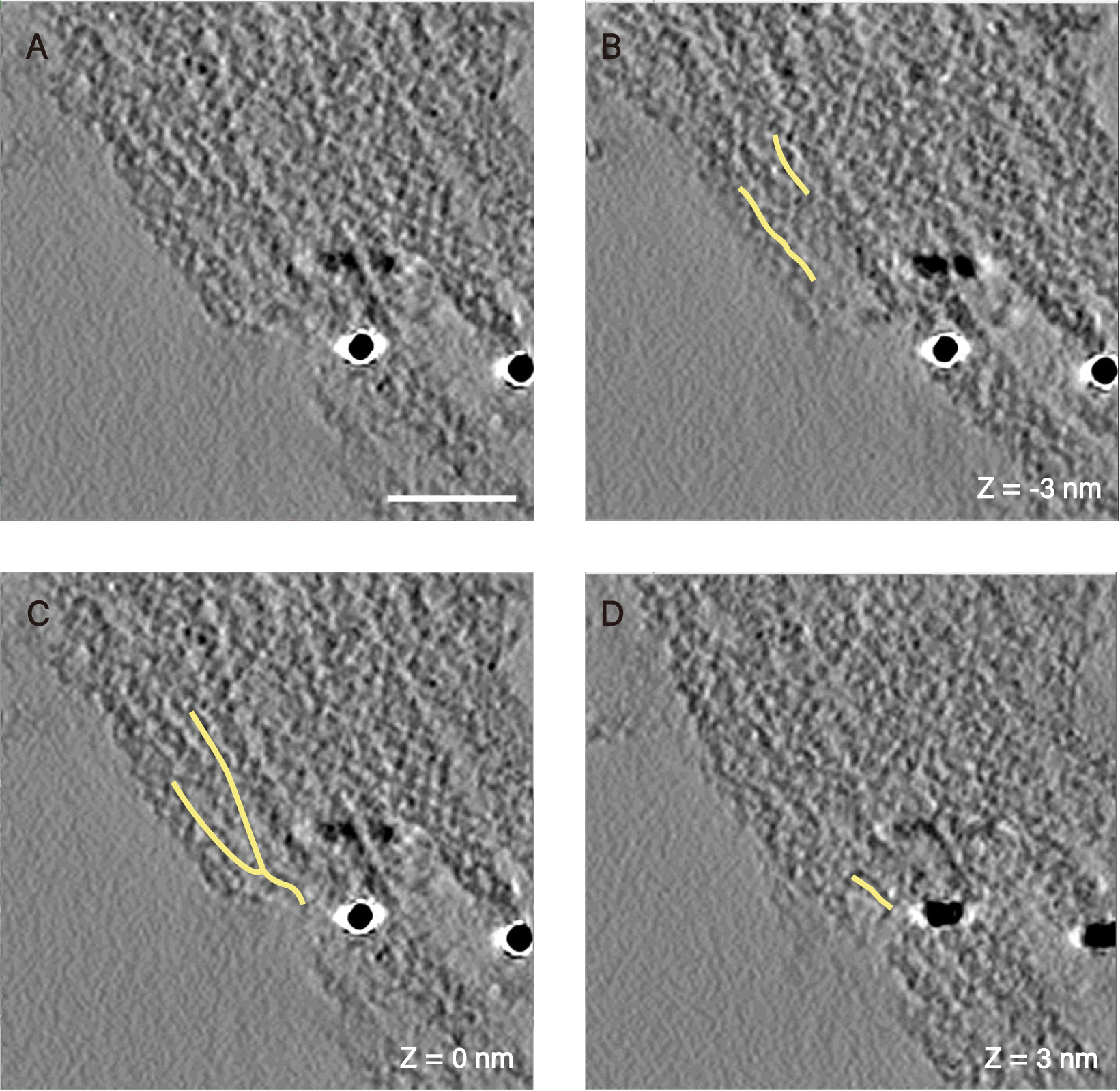


**The branched filament in structural bundle.**

**A.** Representative three-dimensional reconstruction slice of the CTP-state data. **B,C,D.** different Z-axis levels of the **A**. The two filaments branching out are shown in yellow line. Scale bar: 50 nm.

**Supplementary figure S3**

**
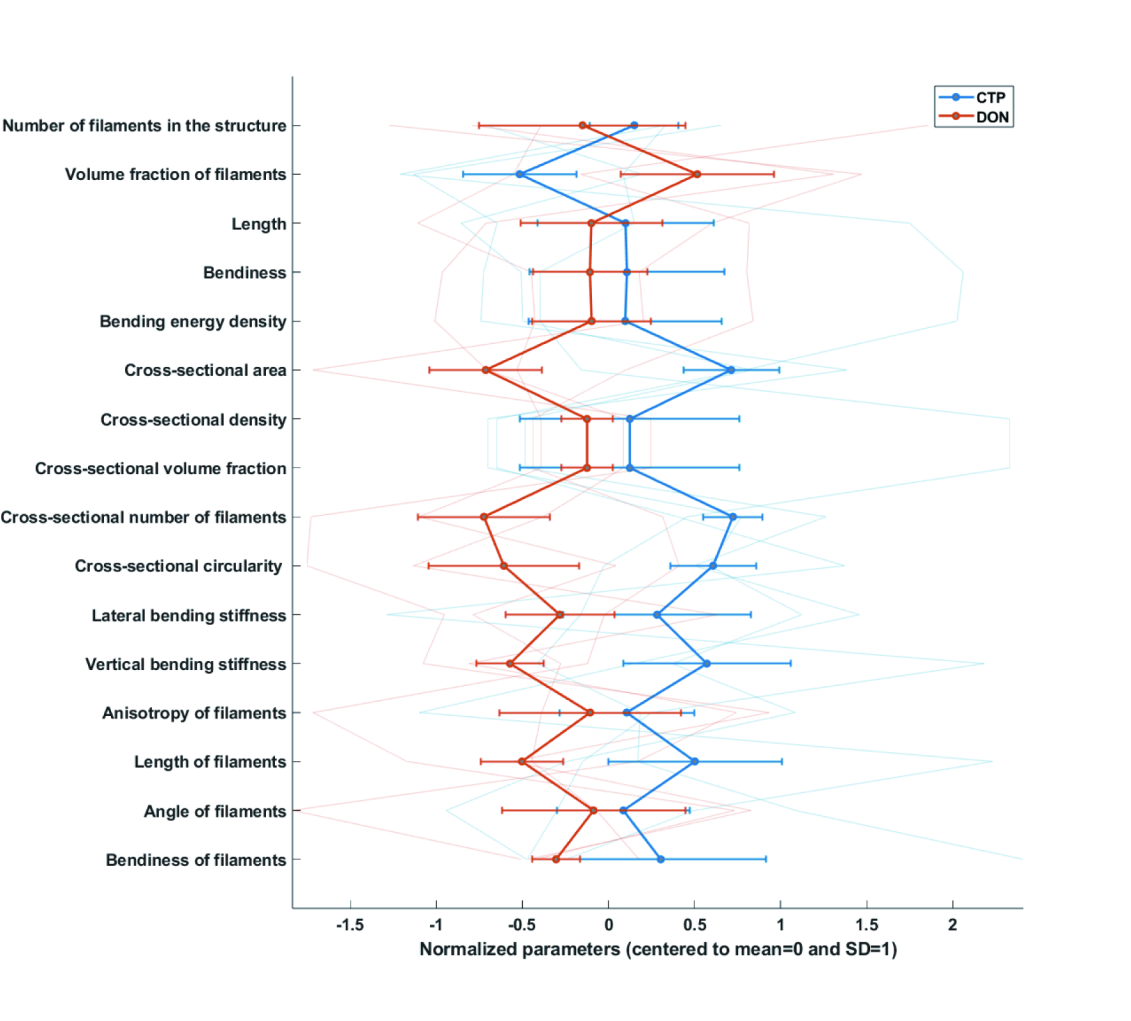
**

**Comparison of multi-parameter normalized quantitative values between the CTP and DON states**

Parameters are normalized by centering them to mean=0 and SD=1. Thus, differences between groups are all relevant and not represented in absolute values. Thick lines indicate the averaged values for all data files in a group, while faint lines show the averaged values of individual data files for every parameter.
